# Supplementary material for: Physical principles of retroviral integration in the human genome
Source: Nat Commun. 2019 Feb 4;10:575. doi: 10.1038/s41467-019-08333-8 (PMC6362086; doi:10.1038/s41467-019-08333-8)
Supplement: Supplementary file 1 — Supplementary Information [file 41467_2019_8333_MOESM1_ESM.pdf]

Physical Principles of Retroviral Integration in the Human Genome: Supplementary Information

D. Michieletto *et al.*

Supplementary Figures

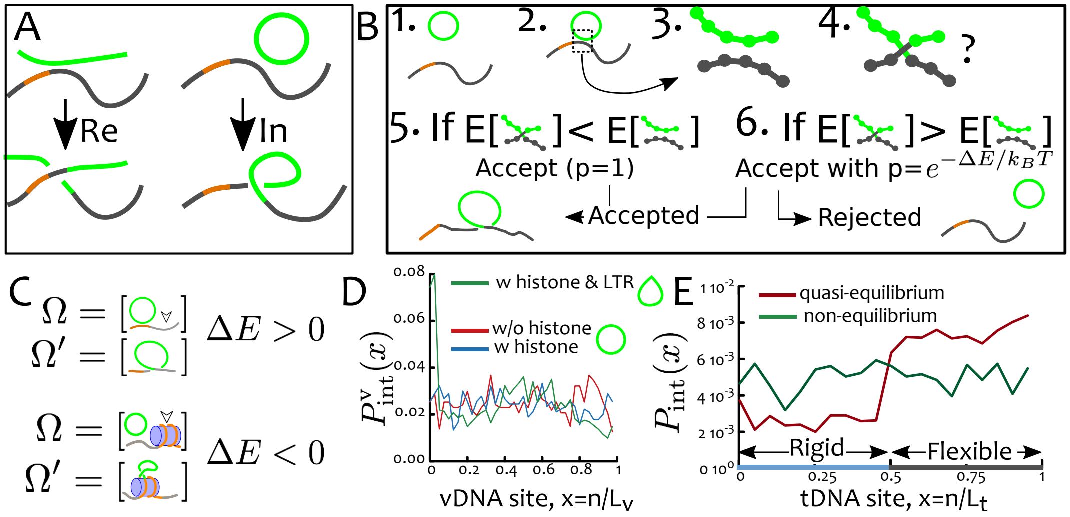

Supplementary Figure **1**: **A** Schematics of recombination and integration events. **B** Model for integration as a quasi-equilibrium process. It can be divided in steps: (1-2) the viral DNA finds a candidate integration site by diffusive search; (3-4) an integration move is performed; (5) the difference in configurational energy is computed and the integration event is accepted if $\Delta E < 0$ or with probability $p = \exp\left[-\Delta E/k_BT\right]$. (6) If rejected the viral DNA can continue its diffusive search for the next candidate. **C** The preference for HIV integration in nucleosomal DNA can be explained by considering this DNA is tightly wrapped and hence heavily bent. For this reason an integration event is likely to reduce the bending of this segment. On the contrary integration in naked DNA must locally increase the bending. **D** Integration probability along the viral DNA. In our simplified model, any site along the viral DNA (of length $L_v = 40\sigma = 294$ bp) can be used as integration point. Considering a more refined model where the first bond along the viral DNA is kinked (thus mimicking the presence of intasome joining the long-terminal repeeats, or LTR, [1]) naturally favours the integration at that site (green line in the plot). At the same time, integration profile in the tDNA is unaltered (see main text). **E** Integration profile in a target DNA segment with heterogeneous flexibility (of length $L_t = 200\sigma = 1470$ bp). The flexible part is naturally favoured by our quasi-equilibrium model since the energy barrier for integration is lower. This is in agreement with experiments [2]. When a purely non-equilibrium model is used, i.e. no ΔE is computed and every event is accepted, the integration probability is uniform. Integrations profiles are obtained by averaging at least 1000 integration events.

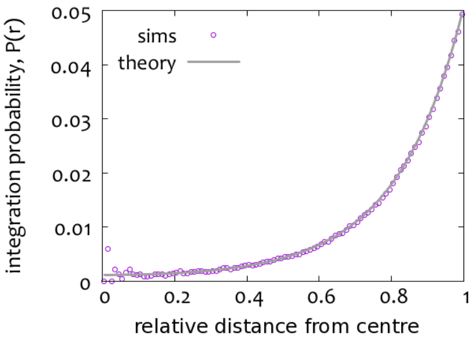

Supplementary Figure **2**: Probability distribution $\rho(r)$. Data points are obtained averaging over $5\,10^5$ random walks starting from the surface of a sphere of radius R and allowed to diffuse and react within the sphere at constant D and κ. The line is the prediction obtained from the analytic solution to the reaction-diffusion problem (see eq. (9)).

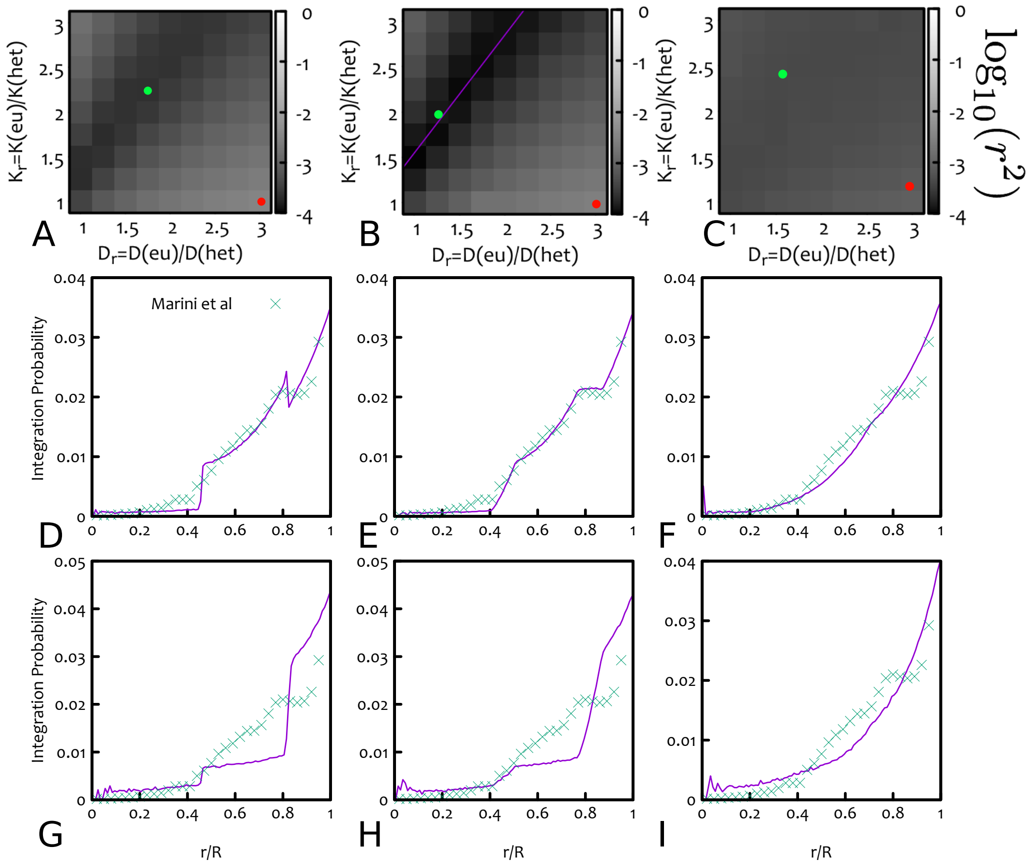

Supplementary Figure **3**: Analysis of the robustness of the results in the parameter space. (**A-C**) We span the 2D space (D_r, K_r) and compute the goodness of fit as $r^2 = \sum_i (o_i - e_i)^2$ (plotted as $\log_{10} r^2$ in grey-scale). From left to right we consider three values of the amplitude $A = 1, 10, 50\sigma$ (or $50, 500, 2500$ nm) which is used to randomly displace the boundary of the three concentric shells for each independent replica of the simulation (average over 10^6 replicas). The green/red dots in the parameter space denote the values for which we find best/worst agreement with experiments. Line in panel **B** is a guide for the eye to identify the region of parameter space where we find a systematically good agreement with experiments. (**D-F**) Examples of best agreements between simulations and experiments for a given boundary displacement. (**G-I**) Examples of worst agreements between simulations and experiments for a given boundary displacement. Experimental dataset is taken from Ref. [3].

Supplementary Note 1: Integration Algorithm

In this section we discuss in more detail the integration algorithm used in this work (also see main text). The integration of viral DNA into the host is a complex process that requires many steps [4]. In our model we summarise this complexity into two main processes: diffusive search of integration site and quasi-equilibrium integration event.

The former process is natural to account for as both target and viral DNAs are polymers diffusing in a noisy environment. Random and diffusive search has been seen in HIV integration in vitro [5] and it is very likely that it also occurs in vivo with even more pronounced complexity given the heterogeneous nuclear composition.

The latter, quasi-equilibrium integration, is our only assumption for the integration event: We start from the observation that HIV integration needs to locally deform the substrate in order to perform the double-strand cleavage and integration; then, we reason that in order to deform the substrate, the integrase enzyme needs to either expend some energy or to exploit thermal fluctuations that facilitate local deformations. In either case, we model this process by computing the total configurational energy immediately before (E) and immediately after (E') the integration move and compute the difference $\Delta E = E' - E$. If $\Delta E < 0$ then this means that the integration move is locally energetically favoured and the move is accepted. On the contrary, if $\Delta E > 0$ we accept it with probability $p = \exp\left[-\Delta E/k_B T\right]$. This effectively mimics thermal fluctuations (of order $k_B T$) that can induce spontaneous local deformations and thus facilitate integration. If the move is rejected the viral DNA resumes the diffusive search (see Suppl. Fig. **1**B for a schematics).

Supplementary Note 2: Integration within Single Nucleosomes

In the first part of our work, we study the integration of viral loops within nucleosomes. The size of viral loops is not a crucial parameter in our model as the reconnection moves occur only "locally", i.e. between two short polymer segments, and irrespectively of the rest of the chain. This is compatible with experiments in vivo and in vitro. Indeed, in the latter case HIV DNA is generally much smaller than in vivo and yet it is still successfully integrated within DNA substrates [6]. For this reason, we consider viral DNA (vDNA) as made of 20 or 40 beads (about 150-294 bp) for computational efficiency.

We model the presence of a histone protein as represented by a sphere of size $\sigma_h = 3\sigma = 7.5$ nm. By assigning an attractive interaction between the histone and a selected segment of the polymer, we can model the wrapping of the DNA around the histone core. This is done using the LJ potential (see Methods in main text) and setting $r_c = 1.8\sigma$ and $\epsilon = 4k_B T$. With this simple model we cannot control the handedness of the wrapping. On the other hand, this is not crucial for our argument. Notice that this model has been used in the literature to describe chromatin reconstitution *in vitro* [7].

Because the integration process is weighted by computing the change in energy before and after the recombination event, the kinetics of integration becomes very slow when we set the persistence length to be $l_p = 20\sigma$. This is because any bending introduced during the recombination heavily contributes to increasing the conformational energy. To speed up the kinetics while maintaining a realistic persistence length, we employ an extensible bond potential, i.e. instead of FENE bonds we use harmonic bonds

$$U^{ab}_{harm}(r) = \frac{\kappa}{2}\left(x - x_0\right)^2 , \tag{1}$$

where $x_0 = 1.1\sigma$ and $\kappa = 20k_BT$. This effectively allows bonds to be more easily stretched and *uniformly* lowers the overall energy barrier along the DNA. Importantly, by using this potential for all bonds along the DNA we are not introducing explicit biases in the integration into any specific region. Any bias in the integration statistics is a result of the presence of histone-like particles and the consequent local larger bending introduced within the nucleosome core (see main text Fig. 1).

The observed bias for nucleosomal DNA can be readily understood by considering the fact that this DNA is tightly wrapped around the histone and hence heavily deformed and storing a large bending energy. This is because the nucleosomal DNA is comparable to DNA's persistence length. If now one imagines using a much larger contour length to wrap the same histone octamer, the resulting configuration is much looser, less bent and thus storing less bending energy. For this reason, one can imagine that an integration event on a segment of nucleosomal DNA lowers the total configurational energy and it is thus an energetically favoured process. on the contrary, integration in naked DNA requires a local deformation that locally increases the bending energy of the tDNA and it is thus energetically disfavoured but possible because of thermal fluctuations (see Suppl. Fig. **1**C for a schematics).

Supplementary Note 3: A Refined Model for Viral DNA with LTR

Up until now, we have considered a model where any segment along the viral DNA can be selected for the integration event. Indeed we observe a uniform integration probability along the viral DNA (vDNA). Although this may seem biologically inaccurate, it is done for computational efficiency as it speeds up the search process. Restricting the integration event to occur in one segment on the vDNA would not change our results.

An improvement in this respect can be done as follows: we can consider a refined model where the viral DNA is now modelled as a loop with a "kink". More specifically, we set a fixed persistence length equal to $l_p = 50$ nm for all the segments of the viral DNA apart from one, where we set $l_p = 0$. By doing so we effectively allow the angle between beads $N, 1, 2$ to assume any sterically accessible conformation without paying any energy penalty. In practice, this translates into the viral DNA often displaying a "kink" at this location. This kink can be seen as the point where the intasome is located and where it keeps the long-terminal-repeats (LTR) together [1].

By performing integrations with this model we observe no change in the integration profile along the target DNA, as expected (indeed it is only the deformation of the substrate that determines the integration profile along the tDNA). Yet we observe a difference in the integration profile along the *viral* DNA. This is because the segment with zero persistence length is more easily deformable with respect to others. For this reason we expect, and observe, a marked increase in integration probability in this segment (see Suppl. Fig. **1**D). See Suppl. Movie 2 for an integration event with kinked vDNA.

Supplementary Note 4: Integration in Naked DNA with Heterogeneous Flexibility

In this section we discuss our results for integrations in substrates with heterogeneous flexibility. We consider a segment of naked DNA 200 beads long (about 1470 bp or 500 nm): the first half of the segment is rigid (persistence length $l_p = 50$ nm) while the second half is flexible (persistence length $l_p = 30$ nm). As one can see in Suppl. Fig. **1**E our simulations show that the integration of a 40 beads (294 bp) viral DNA is favoured in the flexible region, as seen experimentally [2]. This finding can be understood again in terms of energy barriers. The flexible regions allow more easily local deformations that are exploited by the integration machinery to integrate the viral DNA into the substrate.

Importantly, our model can account for this preference and for that shown for nucleosomal DNA with one simple assumption, i.e. that the retroviral integration is a quasi-equilibrium process that needs to overcome an energy barrier to integrate the viral genetic material into the host (through local deformation of the substrate).

Supplementary Note 5: Non-equilibrium Integration

In this section we discuss an alternative integration strategy that is fully non-equilibrium. Here, retroviral integration maintains its diffusive search but it can perform integration within the host without being restricted to the Metropolis test. In other words, any integration move is always accepted. This strategy can be understood as mimicking the fact that an enzyme *may* consume ATP to actively deform the substrate and integrate the viral DNA without the need to wait for thermal fluctuations of the substrate.

We repeat the simulations performed in the previous section (in naked DNA substrates with heterogeneous flexibility) and report our findings in Supplementary Figure **1**E. As one can notice, this time the integration profile is uniform, i.e. the preference towards flexible regions is lost. This finding (flat integration profile in naked DNA with heterogeneous flexibility) is not seen in experiments considering HIV, which instead find a preference for flexible (or curved) DNA [2]. For this reason, we argue that our model for HIV integration as quasi-equilibrium process is a better model for real HIV integration as this gives integration profiles that are

in quantitative agreement with experiments (see main text Fig. 1). Yet, our model predicts that retroviruses that do not require to bend the substrate or that employ ATP to deform the substrate should display integration profiles that are insensitive to the underlying DNA flexibility.

Supplementary Note 6: Integration within Nucleosomal Arrays

In the main text (see main Fig. 2) we consider a model in which several histones form a short reconstituted chromatin fibre. In formulating this model we are inspired by the idea that the chromatin fibre is not a static, crystalline structure but that it is a dynamic, "soft" assembly that minimises energetic costs and that can display heteromophic conformations [8]. Our model is motivated by in vitro [9] and in vivo [10] observations that chromatin assumes a range of possible structures rather than one typical structure.

In our model, the substrate DNA is made of 290 beads with 10 nucleosomal segments 20 beads (160 bp) long interspersed with linker DNA 10 beads long (80 bp). This choice considers a linker DNA slightly longer than the typical one in eukaryotes; yet this accelerates the self-assembly of the chromatin fibre as there is a smaller energy penalty to be paid to loop longer linker DNA.

The reconstitution is simulated by using a model where each of the 10 histones in the system experiences a short-ranged attraction (as before mediated by the LJ potential with $r_c = 1.8\sigma$ and $\epsilon = 4k_BT$) with a specific nucleosomal segment. In other words, for 10 nucleosomal segments and 10 histone-like particles, we assume attractive interactions between each pair (1-1, 2-2, 3-3, ...) and purely steric interactions with all other possible pairs (e.g., 1-2, 2-4, 5-9, ...). Although this is far from realistic (as histones do not have one binding site in the entire genome) we are not here interested in the process of reconstitution *per se* but on the integration statistics within a reconstituted chromatin fibre acting as substrate. For this reason, we employ this simple strategy to reconstitute many (> 1000) chromatin fibres in open, partially folded and condensed states and study the statistics of retroviral integration on these different substrates with varying local chromatin structure (see main text). These states are generated as follows: in the open chromatin state histone-like particles display purely repulsive interactions between one another (i.e. via WCA potential described above with $\sigma_{\text{histone}} = 3\sigma$). Starting from this open state, we then include nearest-neighbour attractions $(i, i \pm 1)$between the histone-like particles: depending on the strength of the attraction this leads to either partially folded fibres (nnp, $r_c = 9\sigma = 3\sigma_{\text{histone}} = 22.5$ nm and $\epsilon = 40k_BT$) or fully condensed fibres (nnf, $r_c = 9\sigma = 3\sigma_{\text{histone}} = 22.5$ nm and $\epsilon = 80k_BT$). Alternatively, we also consider next-nearest-neighbour attractions which lead to zig-zagging fibres (nnn, $r_c = 9\sigma = 3\sigma_{\text{histone}} = 22.5$ nm and $\epsilon = 60k_BT$). These large values of binding affinity are required to stabilise short DNA loops which are formed by the linker DNA (10 beads is half the persistence length of DNA in this model) and overcome steric hindrance of the DNA.

We stress that although this model may be seen as oversimplified, it allows us to reproduce a range of chromatin substrates with conformations that are not too far from the heteromorphic structures seen in vitro or in vivo [10, 8, 9]. More sophisticated and realistic models for chromatin reconstitution employing patchy-particles can in principle be used (see Ref. [7]) but the main physical drivers of retroviral integration, i.e. bending and accessibility, are already fully captured by our simplified model.

Supplementary Note 7: Random Walk Model for Viral Integration in Nuclei

In this section we discuss the random walk model to describe the behaviour of viral DNA entering cell nuclei. We assume that the viral DNA enters the nucleus from the nuclear envelope and begins an unbiased Brownian walk with diffusion coefficient D, i.e. satisfying the Langevin equation

$$\boldsymbol{r}_{t+\Delta t} = \boldsymbol{r}_t + \sqrt{2Dk_BT\Delta t}\boldsymbol{\eta} \tag{2}$$

where $\boldsymbol{\eta}$ is a vector of Gaussian numbers with zero mean and unit variance. Furthermore, we describe the integration process as happening at rate κ. In other words, at each time-step we draw a random number and if it is smaller than $\kappa\Delta t$, we stop the random walk and record its position among the "integrated" viruses.

We average over typically 10^5 random walks starting from positions uniformly distributed on the surface of a sphere of radius R and obtain a distribution of integration events. This distribution is then binned in the radial location r of the integrated event and the count for each bin is divided by the area of the shell at position r, i.e. $4\pi r^2 dr$. This distribution is finally normalised so that its integral over the range $[0, R]$ is unity.

In this model, we take $\sigma = 50$ nm and $R = 200\sigma = 10\,\mu m$. By taking the viscosity of the nucleoplasm at $\eta = 150cP$ we can then obtain $\tau_{br} = 50$ ms. We set $D = 0.05\,\mu m^2/s$, close to the diffusivity of viral capsids in nuclei [11] and choose $\kappa = 0.002\,s^{-1}$ (a parameter largely unknown). The retroviral penetration length is thus $l = \sqrt{D/\kappa} = 5\,\mu m$. Importantly, Eq. (2) can be simulated with spatially varying diffusion coefficient and reaction rates.

Supplementary Note 8: Solution of the Reaction-Diffusion Equation for Viral Integration in the Nucleus – Uniform case

Here we describe the solution of the reaction-diffusion equation

$$\frac{D}{r^2}\partial_r\left(r^2\partial_r\rho(r)\right) - \kappa\rho = 0 \tag{3}$$

with D and κ constants. The equation can be written as

$$\partial_{rr}\rho + \frac{2}{r}\partial_r\rho - \frac{\kappa}{D}\rho = 0\,. \tag{4}$$

By assuming that the solution takes the form of $\rho(r) = r^n f$ we can write

$$\begin{aligned}
&r^n \partial_{rr} f + 2\left[n+1\right] r^{n-1}\partial_r f + \\
&+ \left[2nr^{n-2} + n(n-1)r^{n-2} - \frac{\kappa}{D} r^n\right] f = 0 \\
&r^2 \partial_{rr} f + 2\left[n+1\right] r\partial_r f - \left[+r^2\frac{\kappa}{D} - n(n+1)\right] f = 0\,.
\end{aligned}$$

By setting $n = -1/2$ we find

$$r^2 \partial_{rr} f + r\partial_r f - \left[r^2\frac{\kappa}{D} + \frac{1}{4}\right] f = 0\,, \tag{5}$$

which is the modified Bessel equation

$$x^2 \partial_{xx} y + x\partial_x y - \left[x^2 + m^2\right] y = 0 \tag{6}$$

with $m = 1/2$, $x = r/l$ and $l = \sqrt{D/\kappa}$. The generic solution of this equation is

$$y = C_1 I_m(x) + C_2 K_m(x)\,, \tag{7}$$

with $I_m(x)$ and $K_m(x)$ the modified Bessel functions of order m of the first and second kind respectively. Using this generic form, the steady state distribution $\rho(r) = r^{-1/2} f$ is thus

$$\rho(r) = C_1 \frac{I_{1/2}(r/l)}{\sqrt{r}} + C_2 \frac{K_{1/2}(r/l)}{\sqrt{r}}\,. \tag{8}$$

Here, we note that $\rho(r)$ must not diverge at $r \to 0$; this entails that $C_2 = 0$ and we thus find

$$\rho(r) = \frac{\sinh\left(r/l\right)}{r\,\mathrm{shi(R/l)}} \tag{9}$$

with the normalisation hyperbolic sine integral $\mathrm{shi(t)} \equiv \int_0^{\mathrm{R}} \mathrm{sinh(t)/t\,dt}$ obtained imposing $\int_0^R \rho(r)dr = 1$.

Alternatively, one can define $\rho = f/r$ and find a simple equation for f leading to the following solution of Eq. (4)

$$f = Ae^{-\sqrt{\kappa/D}r} + Be^{\sqrt{\kappa/D}r} \tag{10}$$

with A and B coefficients to be determined. In order for this solution to be well behaved at $r \to 0$ we need $A = -B$, reobtaining Eq. (9).

Supplementary Note 9: Reaction-Diffusion Equation in Heterogeneous Nuclei

In this case we need to solve the system of equations:

$$
\begin{aligned}
D_1\left(\partial_{rr}\rho_1(r)+\frac{2\partial_r\rho_1(r)}{r}\right)-k_1\rho_1(r)=0\\
D_2\left(\partial_{rr}\rho_2(r)+\frac{2\partial_r\rho_2(r)}{r}\right)-k_2\rho_2(r)=0\\
D_3\left(\partial_{rr}\rho_3(r)+\frac{2\partial_r\rho_3(r)}{r}\right)-k_3\rho_3(r)=0
\end{aligned}
\tag{11}
$$

with the continuity conditions

$$
\begin{aligned}
&\rho_1(R_1)=\rho_2(R_1)\ ,\ \rho_2(R_2)=\rho_3(R_2)\\
&\partial_r\rho_1(R_1)=\partial_r\rho_2(R_1)\ ,\ \partial_r\rho_2(R_2)=\partial_r\rho_3(R_2)\\
&\partial_r\rho_1(\epsilon\to 0)=0\,.
\end{aligned}
$$

In these equations, R_1 and R_2 are the position of the boundaries between zones 1-2 and 2-3 respectively. We solve this system of equations with Mathematica `DSolve` which gives

$$
\begin{aligned}
\rho_1 &= \mathcal{N}\frac{\sinh\left(r/l_1\right)}{r}\\
\rho_2 &= \frac{\mathcal{N}}{l_1 r}\left[l_1\sinh\left(\frac{\mathrm{R1}}{l_1}\right)\cosh\left(\frac{r-\mathrm{R1}}{l_2}\right)+l_2\cosh\left(\frac{\mathrm{R1}}{l_1}\right)\sinh\left(\frac{r-\mathrm{R1}}{l_2}\right)\right]\\
\rho_3 &= \frac{\mathcal{N}}{4\kappa_2 l_1 l_2 r}e^{-\frac{r+\mathrm{R2}}{l_3}-\frac{\mathrm{R1}+\mathrm{R2}}{l_2}-\frac{\mathrm{R1}}{l_1}}\left\{\kappa_2\left[l_2 l_3\left(e^{\frac{2\mathrm{R1}}{l_1}}+1\right)\left(e^{\frac{2r}{l_3}}-e^{\frac{2\mathrm{R2}}{l_3}}\right)\left(e^{\frac{2\mathrm{R1}}{l_2}}+e^{\frac{2\mathrm{R2}}{l_2}}\right)+8l_1\sinh\left(\frac{\mathrm{R1}}{l_1}\right)e^{\frac{r+\mathrm{R2}}{l_3}+\frac{\mathrm{R1}+\mathrm{R2}}{l_2}+\frac{\mathrm{R1}}{l_1}}\right.\right.\\
&\left.\left.\left(l_2\cosh\left(\frac{r-\mathrm{R2}}{l_3}\right)\cosh\left(\frac{\mathrm{R1}-\mathrm{R2}}{l_2}\right)-l_3\sinh\left(\frac{r-\mathrm{R2}}{l_3}\right)\sinh\left(\frac{\mathrm{R1}-\mathrm{R2}}{l_2}\right)\right)\right]-D_2\left(e^{\frac{2\mathrm{R1}}{l_1}}+1\right)\left(e^{\frac{2r}{l_3}}+e^{\frac{2\mathrm{R2}}{l_3}}\right)\left(e^{\frac{2\mathrm{R1}}{l_2}}-e^{\frac{2\mathrm{R2}}{l_2}}\right)\right\}
\end{aligned}
$$

where $l_i=\sqrt{D_i/\kappa_i}$ and $\mathcal{N}$ is a normalisation factor that can be set by imposing

$$
\begin{aligned}
\textstyle\int_0^R\left[\Theta(x,0,R_1)\rho_1(r)+\Theta(x,R_1,R_2)\rho_2(r)+\right.\\
\left.\Theta(x,R_2,R)\rho_3(r)\right]dr=1
\end{aligned}
\tag{12}
$$

where $\Theta(x,b_1,b_2)$ is here defined as unity if $b_1\leq x<b_2$ and zero otherwise.

Supplementary Note 10: Resizing Nuclear Shells

In order to obtain quantitative agreement between our model and the data from experiments [3] we observe that we need to reshape the concentric regions in the nucleus of a model T-cell. First we fix the mass (or amount of genetic material) in heterochromatin to be twice the one in euchromatin, i.e.

$$
2\rho_{\mathrm{eu}}V_{\mathrm{eu}}=\rho_{\mathrm{het}}V_{\mathrm{het}}\,. \tag{13}
$$

We further impose that the volume occupied by heterochromatin is the inner and outer layer, whereas the one of euchromatin is the middle one, i.e.

$$
\begin{aligned}
V_{\mathrm{het}}&=\frac{4}{3}\pi R_1^3+\left(\frac{4}{3}\pi R^3-\frac{4}{3}\pi R_2^3\right)\\
V_{\mathrm{eu}}&=\frac{4}{3}\pi R_2^3-\frac{4}{3}\pi R_1^3\,.
\end{aligned}
\tag{14}
$$

In these equations, R_1 and R_2 are the boundaries between the first and second layers and between the second and the third, respectively. We now insert eq. (14) into eq. (13) and can solve for $\rho_{\text{eu}}/\rho_{\text{het}}$:

$$\frac{\rho_{\text{het}}}{\rho_{\text{eu}}} = \frac{2\,(R_2^3 - R_1^3)}{R^3 + R_1^3 - R_2^3}\,. \tag{15}$$

obtaining the equation reported in the main text.

Supplementary Note 11: Robustness in Parameter Space

In this section we discuss the robustness of our theoretical model with respect to the parameter space. Our reaction-diffusion model has two main free parameters: the ratios of diffusion coefficient D_r and integration rate K_r for euchromatin versus heterochromatin. The heterochromatic value of diffusion D_0 is set to be similar to that experimentally measured for the viral capsid in the nucleus [11], i.e. $D_0 = 0.05\mu m^2/s$. The heterochromatic integration rate is, to the best of our knowledge, unknown and we set it to $K_0 = 0.01s^{-1}$ to broadly match the experimental data from Ref. [3]. As explained above, the key parameter here is the "penetration length" $l = \sqrt{D_0/K_0}$ which we find needs to be about $l \simeq \sqrt{5}\mu$m to yield broad agreement with the experimental trend (see main text). Albeit our model with $D_r = 1$ and $K_r = 1$ (i.e. both eu- and heterochromatin have the same D_0 and K_0) is in fair agreement with the data (see main text) the fine details of the experimental curve cannot be captured by these parameters. We then note that while varying D_r and K_r we can obtain a closer agreement, our simulations display sudden jumps in the integration profile that are not found in experiments (see main text and Suppl. Fig. **3**A). We thus reasoned that our model neglected the cell-to-cell heterogeneity in the boundaries' location. For this reason we randomised the location of the shells' boundaries and associated an amplitude A which delimited their maximum displacement. By averaging over many ($\sim 10^7$) independent replicas, we obtained smoother curves that started to closely follow the experiments (see main text).

In particular, one can notice that the goodness of the fit – computed as the sum of the residuals $r^2 = \sum_i (o_i - e_i)^2$, where o_i is the observed (calculated numerically) and e_i the expected (experimental) value – is generally robust across the parameter space but it shows a systematic improvement in a diagonal strip $K_r \simeq 0.25 + 1.3 D_r$ (see Suppl. Fig. **3**B).

Finally, by varying the maximum amplitude of the randomisation of the boundary ($A = 1, 10$ and 50 σ, with $\sigma = 50$ nm are shown in Suppl. Fig. **3**) we show that this parameter affects the qualitative behaviour of the integration profile. Specifically, small values A yield abrupt changes which correspond to the jump from one shell to the next. Yet, large values of A smooth out the curves so much that they nearly recover the case with constant diffusion and integration rate. We find that intermediate values (e.g., $A \simeq 10\sigma = 500$ nm) best match the trend seen in the experimental curve. This is shown in the heat maps of Suppl. Fig. **3**, as the case

with $A = 10\sigma$ displays the lowest values of r^2 in some regions of the parameter space.

We finally comment that the best agreement with data from Ref. [3] is obtained when the integration in constitutive heterochromatin (zone 3, most inner shell) is reduced from that of the one predicted by the reaction-diffusion penetration length. We do this by assigning a small probability ($p = 0.2$) for the virus to enter zone 3. This refinement is motivated by the fact that zone 3 is often populated by the nucleolus. This sub-nuclear structure excludes non-ribosomal DNA and thus should not allow the entry of the vDNA. Again, the stochastic nature of our model in accounting for this exclusion is related to the expected cell-to-cell heterogeneity in the precise location of the nucleolus.

Supplementary References

[1] Lesbats, P. *et al.* Retroviral DNA Integration. *Chem. Rev.* **116**, 12730–12757 (2016).

[2] Pruss, D., Bushman, F. & Wolffe, A. Human immunodeficiency virus integrase directs integration to sites of severe DNA distortion within the nucleosome core. *Proc. Natl. Acad. Sci. USA* **91**, 5913–5917 (1994).

[3] Marini, B. *et al.* Nuclear architecture dictates HIV-1 integration site selection. *Nature* **521**, 227–231 (2015).

[4] Maskell, D. P. *et al.* Structural basis for retroviral integration into nucleosomes. *Nature* **523**, 366–369 (2015).

[5] Jones, N. D. *et al.* Retroviral intasomes search for a target DNA by 1D diffusion which rarely results in integration. *Nat. Commun.* **7**, 1–9 (2016).

[6] Pruss, D., Reeves, R., Bushman, F. & Wolffe, A. The influence of DNA and nucleosome structure on integration events directed by HIV integrase. *J. Biol. Chem.* **269**, 25031–25041 (1994).

[7] Brackley, C. A., Allan, J., Keszenman-Pereyra, D. & Marenduzzo, D. Topological constraints strongly affect chromatin reconstitution in silico. *Nucleic Acids Res.* **43**, 63–73 (2015).

[8] Garces, R., Podgornik, R. & Lorman, V. Antipolar and Anticlinic Mesophase Order in Chromatin Induced by Nucleosome Polarity and Chirality Correlations. *Phys. Rev. Lett.* **238102**, 1–5 (2015).

[9] Grigoryev, S. A. & Woodcock, C. L. Chromatin organization - The 30nm fiber. *Exp. Cell Res.* **318**, 1448–1455 (2012).

[10] Ou, H. D. *et al.* ChromEMT: Visualizing 3D chromatin structure and compaction in interphase and mitotic cells. *Science (80-.).* **357** (2017).

[11] Bosse, J. B. *et al.* Remodeling nuclear architecture allows efficient transport of herpesvirus capsids by diffusion. *Proc. Nat. Acad. Sci. USA* **112**, E5725–E5733 (2015).
